# Towards PDT with Genetically Encoded Photosensitizer KillerRed: A Comparison of Continuous and Pulsed Laser Regimens in an Animal Tumor Model

**DOI:** 10.1371/journal.pone.0144617

**Published:** 2015-12-11

**Authors:** Marina Shirmanova, Diana Yuzhakova, Ludmila Snopova, Gregory Perelman, Ekaterina Serebrovskaya, Konstantin Lukyanov, Ilya Turchin, Pavel Subochev, Sergey Lukyanov, Vladislav Kamensky, Elena Zagaynova

**Affiliations:** 1 Nizhny Novgorod State Medical Academy, Nizhny Novgorod, Russia; 2 Lobachevsky State University of Nizhny Novgorod, Nizhny Novgorod, Russia; 3 Shemyakin-Ovchinnikov Institute of Bioorganic Chemistry of the Russian Academy of Sciences, Moscow, Russia; 4 Institute of Applied Physics of the Russian Academy of Sciences, Nizhny Novgorod, Russia; 5 Pirogov Russian National Research Medical University, Moscow, Russia; AntiCancer Inc., UNITED STATES

## Abstract

The strong phototoxicity of the red fluorescent protein KillerRed allows it to be considered as a potential genetically encoded photosensitizer for the photodynamic therapy (PDT) of cancer. The advantages of KillerRed over chemical photosensitizers are its expression in tumor cells transduced with the appropriate gene and direct killing of cells through precise damage to any desired cell compartment. The ability of KillerRed to affect cell division and to induce cell death has already been demonstrated in cancer cell lines *in vitro* and HeLa tumor xenografts *in vivo*. However, the further development of this approach for PDT requires optimization of the method of treatment. In this study we tested the continuous wave (593 nm) and pulsed laser (584 nm, 10 Hz, 18 ns) modes to achieve an antitumor effect. The research was implemented on CT26 subcutaneous mouse tumors expressing KillerRed in fusion with histone H2B. The results showed that the pulsed mode provided a higher rate of photobleaching of KillerRed without any temperature increase on the tumor surface. PDT with the continuous wave laser was ineffective against CT26 tumors in mice, whereas the pulsed laser induced pronounced histopathological changes and inhibition of tumor growth. Therefore, we selected an effective regimen for PDT when using the genetically encoded photosensitizer KillerRed and pulsed laser irradiation.

## Introduction

Photodynamic therapy (PDT) is a method for the treatment of oncological and some non-oncological diseases based on killing pathologic cells as a result of the production of reactive oxygen species (ROS) by a photosensitizer under exposure to light. A typical photosensitizer is a chemically synthesized cyclic tetrapyrrole administered into the body intravenously or topically. The main problems with chemical photosensitizers are associated with their heterogeneous distribution within the lesion and cells, their redistribution over time, and nonspecific drug accumulation in the skin and mucosae. This has encouraged a search for more efficacious phototoxic drugs and modified treatment regimens.

As soon as the first phototoxic protein KillerRed (KR) was engineered in 2006 [1], the idea arose that it could be used as a genetically encoded photosensitizer for very specific direct cell killing. The main advantages of KR over chemical photosensitizers are its expression in tumor cells transduced with the appropriate gene and direct killing of cells through precise damage to the targeted cell compartment.

KR is a dimeric red fluorescent protein (Ex-max 585 nm, Em-max 610 nm) that generates ROS upon irradiation with light [1]. The structural basis for its phototoxicity is a water-filled channel facilitating access to the chromophore and the presence of two key reactive residues Glu68 and Ser119, adjacent to the chromophore [2, 3]. There is evidence that KR produces ROS via a Type I photosensitizing mechanism [4]. The photosensitizing reaction is thought to be associated with the formation of a dianionic radical chromophore and the subsequent transfer of an electron to O_2_ to generate superoxide, which is accompanied by the bleaching of the chromophore [3, 5].

The ability of KR to initiate cell death in a light-mediated manner was first demonstrated by Bulina et al. on cultured bacterial and cancer cells [1]. The use of KR as an optogenetic tool [6, 7] and photosensitizer for PDT was proposed [8].

It has been shown in mammalian cell cultures that the mechanism of cell death depends especially on the intracellular location of the KR. For example, KR expressed in mitochondria induced apoptosis after illumination [1]. With KR localized in the plasma membrane the cells died through necrosis [9], while for KR located in a lysosome the cell death pathway depended on the light intensity and dose [10]. When KR was expressed in nuclei, exposure to light led to a blockage of cell division [6].

In our earlier studies we found that KR can be used to damage cancer cells *in vivo*. Substantial dystrophic cellular changes were observed in KR-expressing HeLa tumors inoculated subcutaneously into immunodeficient mice [8]. However, to achieve a cytotoxic effect a rather intensive treatment regimen was required: the tumors were exposed to illumination with a yellow continuous wave (CW) laser (593 nm, 150 mW/cm^2^, 270 J/cm^2^) daily for 7 days, which was only really feasible on slowly growing tumors. It is apparent that any further development of this approach for PDT would require optimization of the treatment regimen and the testing of it on other tumor models, including those transplanted into immunocompetent mice.

The current work was aimed at the selection of the treatment regimen for PDT of CT26 mouse tumors expressing KR. The treatment parameters were selected such that they would cause satisfactory photobleaching of the KR in subcutaneous tumors without any excessive temperature effects on the skin surface. The PDT was implemented at 593 nm, 150 mW/cm^2^, 270 J/cm^2^ for the CW laser, and at 584 nm, 225 mW/cm^2^, 337 J/cm^2^ for the pulsed laser on the days 6, 7, and 8 of tumor growth. Histological examination of the tumor tissue was performed on the day following the last irradiation and tumor growth was followed for a further two weeks.

## Materials and Methods

### Cell cultures

CT26, murine colon carcinoma, stably expressing KR in fusion with histone H2B (CT26-KR) and a non-expressing counterpart were used. The cell lines were cultured in DMEM with 10% FCS, 1% glutamine, 10 units/mL penicillin and 10 μg/mL streptomycin. The cells were collected for injection by adding 1 mL of trypsin-EDTA (25%) to the 25 mm^2^ plate for 5 min at 37°C.

The CT26-KR cell line was obtained by lentiviral transduction. For lentiviral transduction a NheI-blunt PCR fragment containing the H2B–tKR open reading frame was cloned into a NheI- and EcoRV-digested pRRLSIN.EF1.WPRE vector with a modified multiple cloning site. The vector was kindly provided by Prof. Didier Trono (E´cole Polytechnique Fe´de´ rale de Lausanne, Lausanne, Switzerland). The lentiviral particles for mammalian cell infection were obtained according to standard procedure. For lentiviral infection, CT26 cells were plated on d = 35 mm cell culture dishes (SPL Life sciences, Korea) at a density of 2.5х10^4^ cells/dish in DMEM with 10% FCS, 1% glutamine, 10 units/mL penicillin and 10 *μ*g/mL streptomycin. After 24 h culturing, the medium was changed for the medium with viral particles.

### Analysis of fluorescence of CT26-KR cells

The fluorescence of the infected cells was analyzed 5–7 days post–infection, using flow cytometry. For flow cytometric analysis, cells were washed with PBS and re-suspended to the final density of 2 x 10^5^ cells/mL. Analysis was carried out using Cytomics FC500 flow cytometer, equipped with an air-cooled argon-ion laser operating at 488 nm (Beckman Coulter). The following detection parameters were used: 6 mW laser power and 620 nm band pass filter (FL3 channel). A minimum of 5000 events were collected for each sample. The portion of the cell population with the highest fluorescence intensity was selected with FACS (brightest 30%).

For sterile cell sorting, 2×10^6^ cells were re-suspended in PBS with 5% FCS at a density of 5×10^5^ cells/mL. The cell suspension was then filtered through a 70 *μ*m nylon mesh cell strainer. Using a MoFlo cell sorter (DakoCytomation), with a minimum of 1.5×10^5^ events being collected into a sterile 2 mL tube containing DMEM, 10% (v/v) FBS, 10 units/mL penicillin and 10 *μ*g/mL streptomycin.

Live cell imaging was performed in HEPES-buffered MEM (Sigma) supplemented with 10% (v/v) FCS at 37°C in 5% CO_2_ atmosphere. For fluorescence microscopy, a Leica AF6000 LX imaging system, based on a DMI 6000 B inverted microscope equipped with a Photometrics Cool SNAP HQ CCD (charge-coupled device) camera, was used. A 120W HXP short arc lamp (Osram) was used as a light source. A standard S Blue filter set [excitation D 405/10x nm, emission D 460/50 nm] and Tx2 filter set [excitation BP 560/40 nm, emission 645/75 nm] were used to acquire blue and red fluorescence, respectively.

### Tumor model

The experiments were conducted on female BALB/c mice weighing 18–20 g. For tumor generation, the animals were challenged subcutaneously with 5x10^5^ CT26 or CT26-KR cells in 100 μL PBS in the right flank. All experimental procedures were approved by the Ethical Committee of the Nizhny Novgorod State Medical Academy (Russia).

### Fluorescence imaging in vivo

Fluorescence imaging of tumors was performed *in vivo* using a molecular imaging system, an IVIS-Spectrum (Caliper Life Sciences, USA) or a custom-built back-reflectance imaging setup (Institute of Applied Physics RAS, Russia) [8]. In the IVIS-Spectrum, fluorescence was excited at a wavelength of 570/30 nm and recorded at 620/20 nm. In the custom-built imaging setup, a 585 nm LED was used for excitation, while emission was detected in the using 685/80 nm filter. The signal linearity of both imaging systems was tested using a calibrated light source and it was found to be sufficiently high within the measurable dynamic range. The fluorescence images were analyzed using LivingImage or ImageJ 1.39p software. The average fluorescence intensity of each tumor was calculated at different time-points, and expressed as a percentage decrease, relative to the initial value measured before irradiation.

### PDT treatment

A diode pumped solid state yellow laser (MGL, Changchun New Industries Optoelectronics Tech. Co., Ltd. (CNI) P.R. China) with a 593 nm wavelength was used for PDT in CW mode. For the pulsed mode, tumors were irradiated by a tunable pulsed laser (LS-2214PC, LOTIS TII, Belarus) at a wavelength of 584 nm, 15 ns, 10 Hz. A small part of light energy was directed to a pyro sensor (ES11C, Thorlabs, USA) to measure the energy of each laser pulse.

To select the laser treatment parameters for PDT, the extent of photobleaching of the KR in the tumors and the temperature on the tumor surface were assessed. Fluence rates of 110, 150, and 260 mW/cm^2^ for the CW laser and 225 mW/cm^2^ for the pulsed laser were tested using exposure times from 5 to 30 min. Similar treatment regimens had previously been tested on 3D tumor spheroids [11]. Before and during the irradiation, the skin surface temperature was measured using an IR thermograph (CEM-ThermoDiagnostics, CEMTechnology, Russia). The groups of mice treated in these regimens each consisted of 3 animals bearing CT26-KR tumors 5–6 mm in diameter (6–7 days after cancer cell injection).

For the PDT experiment, tumor-bearing animals were divided into 6 groups of 10 animals: “CT26, CW”, “CT26, pulsed”, “CT26-KR, CW”, “CT26-KR, pulsed”, “CT26, No treatment”, and “CT26-KR, No treatment”. A further 3 mice with tumors were included in both the untreated “CT26” and the “CT26-KR” groups. The tumors were irradiated on the 6, 7, and 8^th^ days of growth at a fluence rate of 150 mW/cm^2^ and a light dose of 270 J/cm^2^ for the CW mode, or at 225 mW/cm^2^ and 337 J/cm^2^ for the pulsed mode. 24 hours after PDT, 3 randomly selected tumors in each treated, and 6 in each untreated, group were excised and fixed in 10% neutral buffered formalin for subsequent histopathology. Other tumors were monitored for 2 weeks—their size was measured with a caliper twice a week. The tumor volume was calculated as a*b*b/2, where *a* is the length and *b* is the width of the tumor.

### Histopathology

Formalin fixed tissue specimens were dehydrated, embedded in paraffin, cut into 4 μm sections and stained with hematoxylin and eosin (H&E). The cancer cells in the slides of each tumor were counted in 5 randomly selected microscope fields of 0.01 mm^2^ at 400x magnification. The percentages of the unaltered (typical) tumor cells, including mitotic figures, and of altered cells were calculated. The altered cells included the any with dystrophic changes (swollen hyperchromic nuclei, vacuolated cytoplasm, and chromatin condensation) and cells with any indication of apoptosis.

### Statistics

Mean ±SD values were used for the expression of data. Statistical differences between groups were determined by one-way ANOVA with the Bonferroni post-hoc test. *P*≤0.05 was considered statistically significant.

## Results

### Fluorescence of CT26-KR cell line

**Fig 1:**
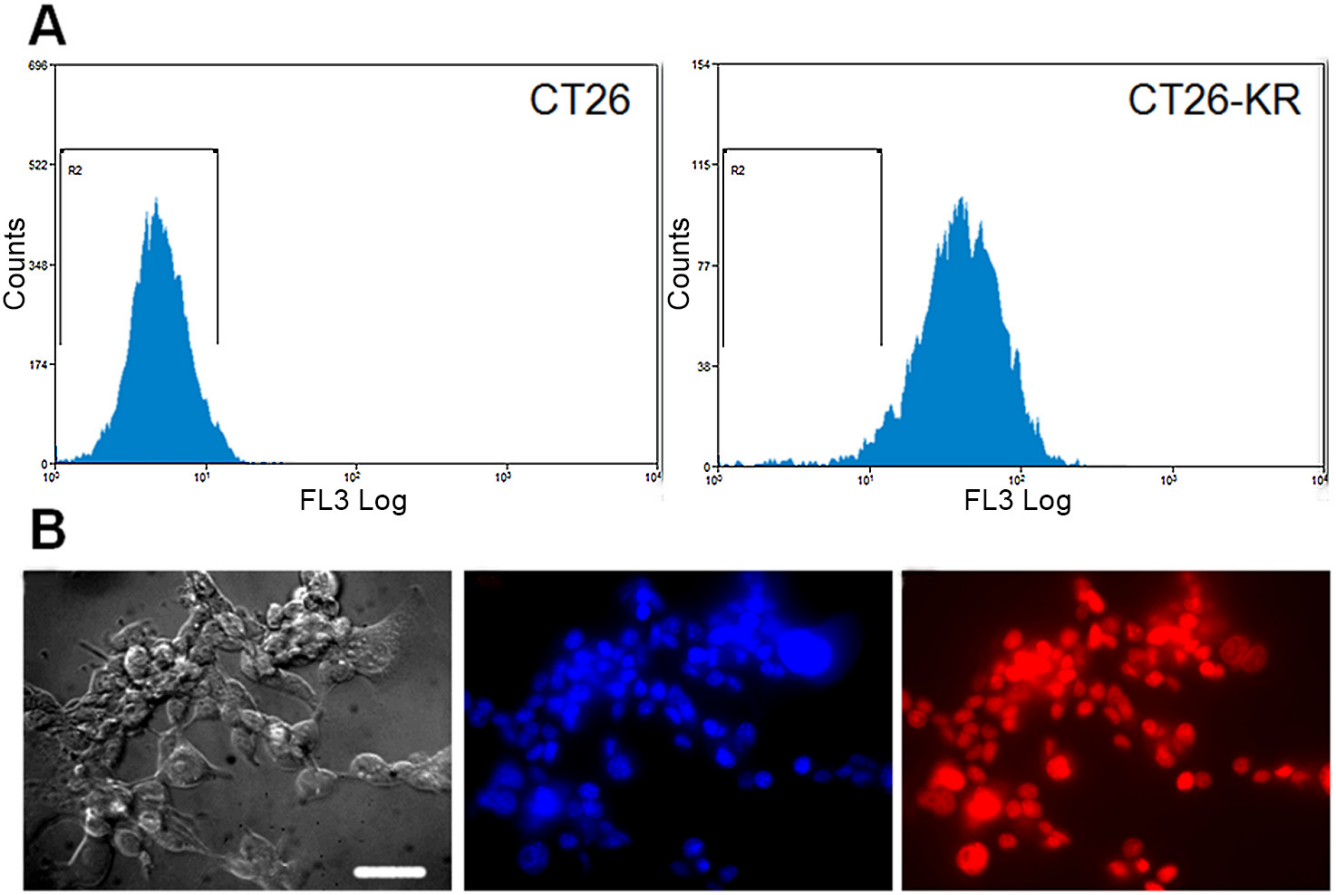
Fluorescence of CT26-KR cell line. A) Flow cytometry of CT26 (R2 = 97.3%) and CT26-KR (R2 = 4.6%) cells. B) Fluorescence microscopy of CT26-KR cells: transmitted light, blue fluorescence of Hoechst 33342, red fluorescence of KR. Bar is 50 μm.

The CT26 cell line stably expressing KR was obtained by lentiviral transduction and its fluorescence was analysed using flow cytometry and fluorescence microscopy. Flow cytometry showed less than 5% non-fluorescent cells in CT26-KR cell line (Fig 1A). This result correlates well with microscopic analysis where all cell nuclei, stained by Hoechst 33342, also display red fluorescence of KR (Fig 1B).

### Photobleaching of KR in tumors and thermal effects

It is known that photobleaching of KR accompanies the photosensitization reaction [1, 8, 11], and can potentially predict the efficiency of PDT. Another factor accompanying PDT is the potential change in tumor temperature due to light delivery [12, 13]. To avoid any temperature effects on the results of PDT with KR, it was important to select treatment parameters allowing maximum photobleaching of the photosensitizer to be achieved in the tumors with minimal increase in temperature.

**Fig 2:**
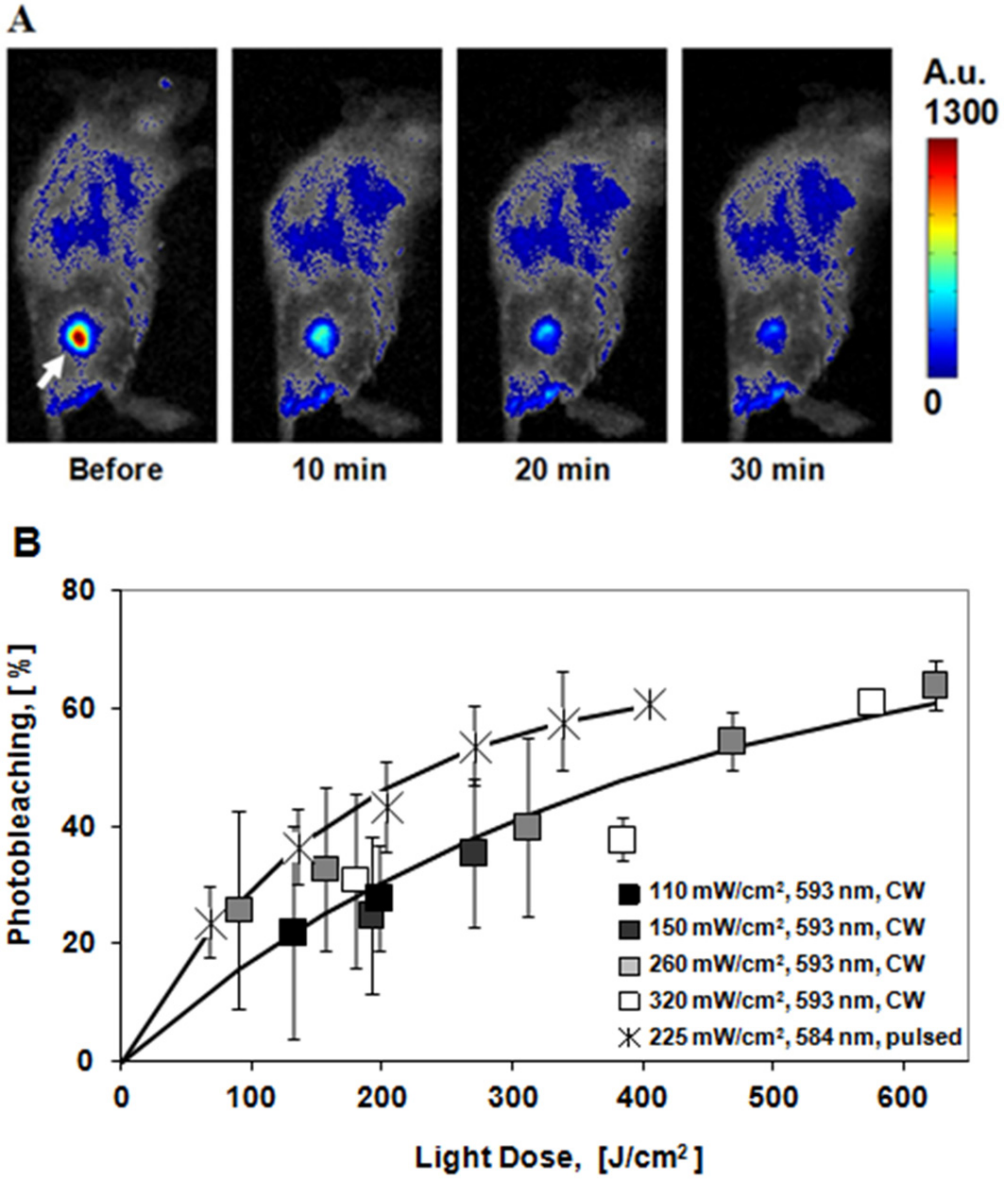
Photobleaching of KR in CT26-KR tumors. A) Fluorescence imaging of CT26-KR tumor *in vivo* during irradiation with the pulsed laser at 225 mW/cm^2^. Tumor is shown by the arrow. B) Photobleaching as a function of light dose for the five fluence rates. The results are expressed as mean ± SD (n = 3). The solid lines show exponential approximations (*R* ^*2*^ = 0.91 and 0.99 for the CW and pulsed laser modes, respectively).

Fig 2 shows that the fluorescence of the tumors decreased in the process of irradiation due to KR photobleaching. For a range of fluence rates 110–320 mW/cm^2^ delivered in the CW mode there was no difference in the bleaching rates, whereas the bleaching rate was higher in pulsed mode. The dependences of fluorescence intensities on the light dose were found to be best fit to single exponential curves. At high light doses, the decrease in fluorescence intensity was ~60% for both, CW and pulsed, modes.

Exposure of the tumors to the CW laser increased the temperature of the skin surface above the tumor from 31.7 to 35.1°C, 36.9°C, and 39.5°C at 150, 260, and 320 mW/cm^2^, respectively (Table 1). Owing to the significant temperature rises produced when using illumination with high fluence rates, treatments at 260 and 320 mW/cm^2^ were excluded from further consideration. No temperature effects were detected upon illumination at 110 mW/cm^2^ in CW mode and at 225 mW/cm^2^ in pulsed mode. However, a fluence rate of 110 mW/cm^2^ caused poor bleaching of KR (~25%), that would most probably be insufficient to destroy the cancer cells.

**Table 1:** Temperature on the CT26-KR tumor surface after laser treatment.

Regimen	110 mW/cm^2^, CW	150 mW/cm^2^, CW	260 mW/cm^2^, CW	320 mW/cm^2^, CW	225 mW/cm^2^, pulsed
**t, °C**	31.4±0.9	35.1±0.9	36.9±0.2	39.5±1.2	30.7±0.5
**Δt, °C**	0	2.1±0.9	6.0±0.3	7.3±0.8	0

The baseline temperature (before irradiation) was 31.7±1.3°C.

Therefore, based on the fluorescence and temperature measurements, two regimens were selected for the PDT: 270 J/cm^2^ (150 mW/cm^2^, 30 min) in CW mode with a 593 nm wavelength, and 337 J/cm^2^ (225 mW/cm^2^, 25 min) in pulsed mode at 584 nm.

### Phototoxicity of KR in tumors

**Fig 3:**
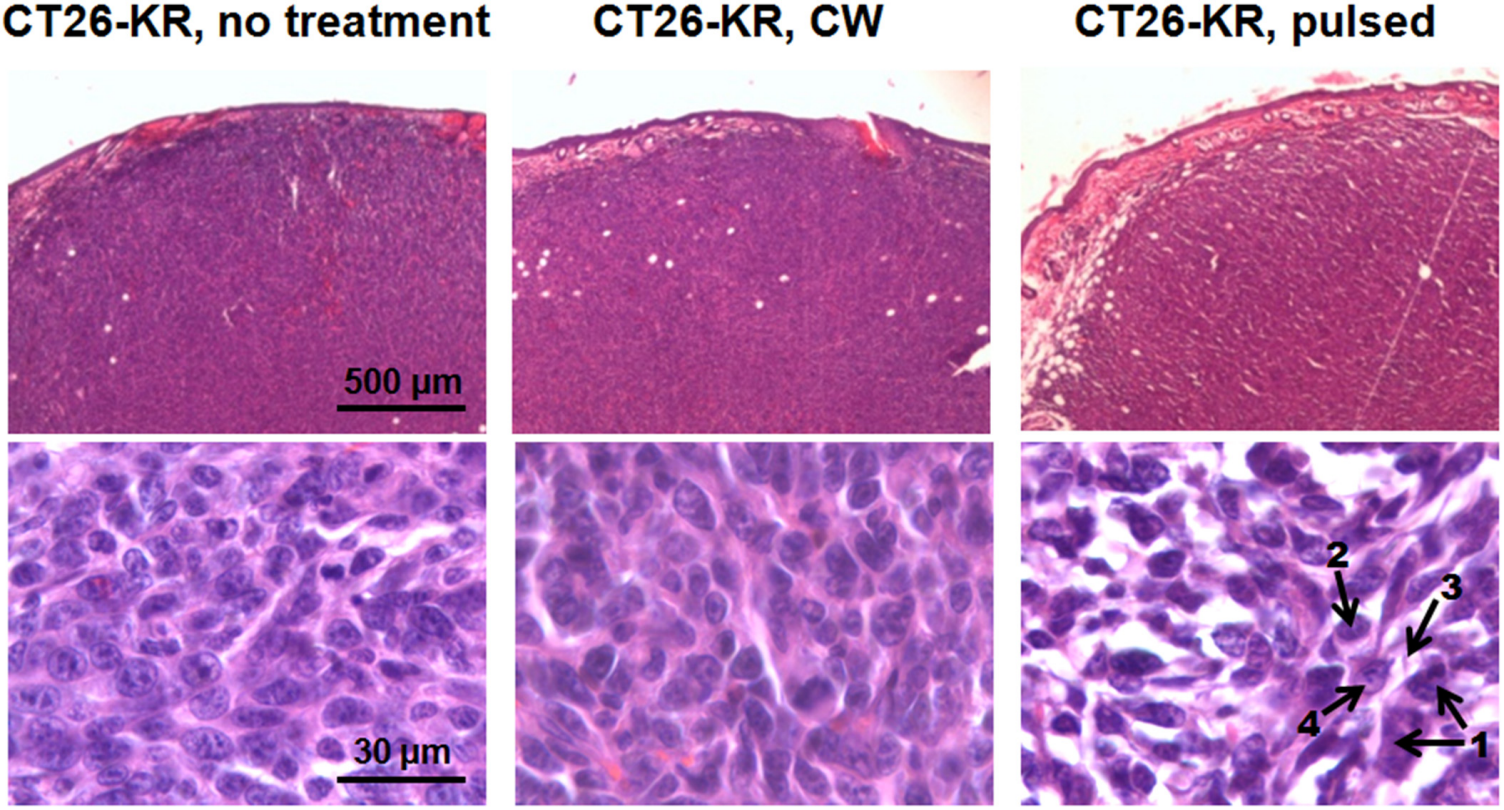
A histological view of CT26-KR tumors 24 hours after PDT with CW or pulsed lasers and untreated control. Representative tissue sections stained with H&E are shown. The cellular disorders induced by PDT in pulsed mode are shown by the numerated arrows: 1—swollen hyperchromic nuclei, 2—chromatin condensation, 3—vacuolated cytoplasm, 4—apoptosis hallmarks.

PDT of KR expressing tumors performed from the 6^th^ to 8^th^ days of tumor growth with the CW laser (150 mW/cm2, 30 min, x3) did not induce any abnormalities in the histopathology (Fig 3).

The tumor tissue had a dense structure and consisted of polymorphic cells of different sizes with large round or oval nuclei containing diffusively distributed chromatin and 1–2 nucleoli. The slightly basophilic cytoplasm formed a thin ring around the nucleus. The CT26-KR tumors treated in CW mode were histopathologically identical to the untreated specimens as well as to the treated and untreated CT26 tumors.

Irradiation of CT26-KR tumors with the pulsed laser (225 mW/cm^2^, 25 min, x3) resulted in pronounced dystrophic changes in the cancer cells (Fig 3). The cellular abnormalities included vacuolated cytoplasm, swollen or irregular hyperchromic nuclei and chromatin condensation. Calculating the dystrophically changed cells showed that their percentage increased from 17.6% in the CT26-KR untreated group to 62.8% after PDT using pulsed mode (Table 2). Among the cellular disorders, swelling of the nuclei and vacuolization of the cytoplasm contributed the most. Correspondingly, the proportion of separately counted, unaltered cells decreased from 82.4% to 37.2%. Besides inducing dystrophic changes in the cells, PDT with KR in pulsed mode inhibited mitosis (0.2% vs. 8.5% cells with mitotic figures) and activated apoptosis (8.4% vs. 0.2% cells with apoptotic hallmarks). Since most of the treated cells were enlarged, the number of cells in the field of view decreased.

**Table 2:** Quantification of the cellular disorders induced by PDT with KR.

	CT26-KR			CT26		
	CW	Pulsed	No treatment	CW	Pulsed	No treatment
**Unaltered tumor cells, %**	**79.9±2.4**	**37.2±2.2** *	**82.4±0.7**	**80.0±4.8** ^#^	**80.5±0.8**	**83.8±1.0**
Mitosis figures, %	6.9±1.8	0.2±0.1*	8.5±1.6	7.1±2.2	10.1±0.3	9.3 ±1.4
**Altered tumor cells, %**	**20.1±2.4**	**62.8±2.2** *	**17.6±0.7**	**20.0±4.8** ^#^	**19.5±0.8**	**16.2±1.0**
Swollen hyperchromic nuclei, %	13.5±1.4	35.8±2.3*	10.6±2.6	11.5±3.1	12.6±0.9	10.9±1.3
Vacuolated cytoplasm, %	12.9±3.4	30.8±1.0*	10.9±1.1	10.7±3.5	10.8±0.9	10.4±1.8
Chromatin condensation, %	2.0±0.8	7.0±0.6**	1.4±0.9	5.8±3.6^#^	2.1±1.2	1.1±0.9
Apoptosis hallmarks, %	0.9±0.1	8.4±1.8*	0.2±0.1	0.9±0.3	0.2±0.1	0.3±0.1
**Total number of cells in the field of view**	**145.5±11.4**	**125.1±10.9** **	**137.6±13.2** ^#^	**137.9±12.6**	**144.2±8.6**	**147.9±10.1**

PDT was conducted at 260 J/cm^2^ (150 mW/cm^2^, 30 min) in CW mode or at 337 J/cm^2^ (225 mW/cm^2^, 25 min) in pulsed mode on days 6, 7, and 8 of tumor growth. Analysis was performed 24 hours after irradiation. Each number represents mean ± SD. The percentage of the cells with different morphological signs was calculated in 5 randomly selected fields of view for each tumor.

*, P ≤ 0.01, compared with all other groups.

**, P ≤ 0.03, compared with “CT26-KR, CW” and “CT26-KR, No treatment” groups.

^**#**^, P ≤ 0.01, compared with “CT26, No treatment” group.

PDT with KR in CW mode appeared to have no impact on the tumor cells. The small increase in the number of altered cells (from 17.6% to 20.1%) was similar to that in the “CT26, CW” group (Table 2) and can be attributed to thermal effects or to the photoactivation of endogenous chromophores.

**Fig 4:**
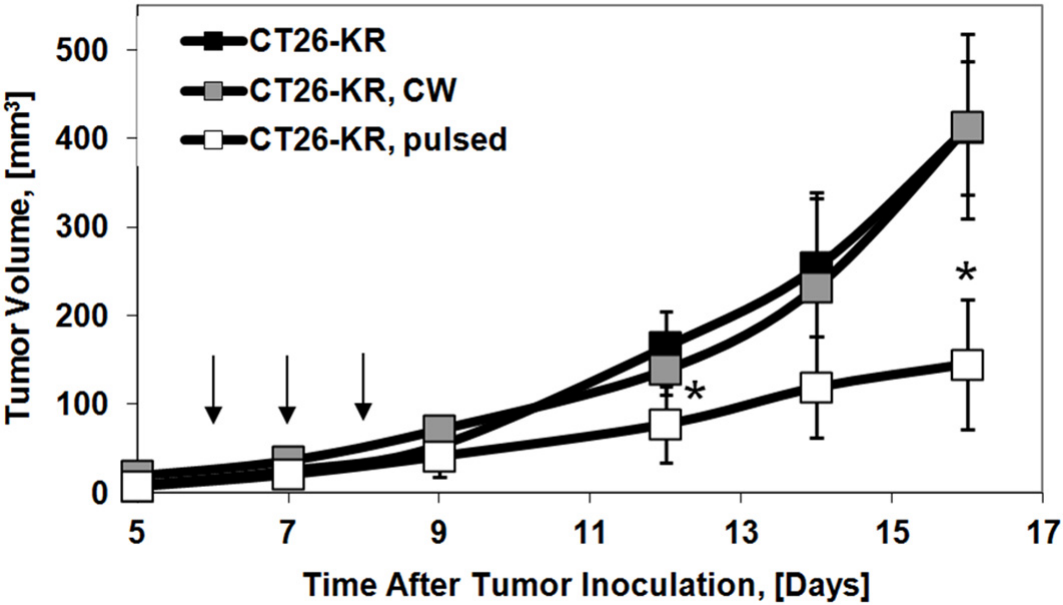
Effect of PDT with KR on the growth of CT26 tumors in BALB/c mice. Mean ± SD (n = 7). PDT was conducted on days 6, 7, and 8 (shown by arrows) after cancer cell inoculation. The tumors were irradiated at 260 J/cm^2^ (150 mW/cm^2^, 30 min) in CW mode or at 337 J/cm^2^ (225 mW/cm^2^, 25 min) in pulsed mode. *, P ≤ 0.01, compared with the control “CT26-KR, No treatment” group.

PDT of the CT26-KR tumors in pulsed mode led to inhibition of tumor growth in mice (Fig 4). Although none of the tumors was completely cured, the growth rate in the “CT26-KR, pulsed” group became slower compared to that in the “CT26-KR, No treatment” group, resulting in significant differences in the tumor sizes by day 16. Irradiation of the CT26-KR tumors with the CW laser had no influence on their growth rate.

## Discussion

The proteins of the green fluorescent protein (GFP) family are thought to be non-phototoxic (at least, under short-term illumination in visible range), and therefore, used in PDT research for noninvasive visualization of tumor response [14, 15] and investigation of the signaling mechanisms [16, 17] without any impact on the results of the therapy. Previous studies on phototoxicity of GFPs showed the low efficiency for triplet state formation and singlet oxygen production by EGFP [18]. Nevertheless, the experiments by Momiyama et al. [19] and Kimura et al. [20] indicated that fluorescent proteins GFP and RFP increased the efficacy of ultraviolet C on cancer cells and, consequently, enhanced PDT.

KR is the only GFP-like protein that displays strong phototoxicity, exceeding that of EGFP by at least 1,000-fold. This property allows it to be considered as a potential genetically encoded photosensitizer for the PDT of cancer.

In this research we examined the effects of both CW and pulsed lasers on KR-expressing colon carcinoma CT26 in mice, and selected treatment regimen effective from the point of view of antineoplastic action. Significant cellular damage and inhibition of tumor growth was caused in CT26-KR tumors by irradiation with the pulsed laser at 337 J/cm^2^ at a wavelength of 584 nm.

The immediate question regarding KR as a therapeutic agent is the best method of delivery of genetic material encoding the protein, or of the protein itself, to the tumor. A small number of systems for KR plasmid delivery to cancer cells have been developed—an adenovirus-mediated system [21], the use of a polymeric micelle-encapsulating quantum dots [22], and the use of chitosan complexes each with a negatively-charged poly(γ-glutamic acid) [23], but at the moment these authors' papers have only reported expression of KR in the gene-transfected cells *in vitro* with a resulting suppression of cell proliferation and viability following irradiation with light. Very recently Tseng et al. reported on a high-efficiency transfection with the KR gene of an aggressive lung tumor model after a single systemic administration using a highly pH-sensitive, negatively charged, polymeric carrier [24]. Another strategy is the targeted delivery of the KR protein to cancer cells. Serebrovskaya et al. constructed an immunophotosensitizer, consisting of a specific anti-p185^HER-2-ECD^ antibody fragment 4D5scFv fused with KR that showed fine targeting properties and efficiently killed p185^HER-2-ECD^-expressing cancer cells upon irradiation with light [25].

Photobleaching-based dosimetry represents a simple and low-cost approach to control of PDT dosage. The ability of a photobleaching-based PDT dose metric to predict the efficacy of PDT has been demonstrated for many chemical sensitizers [26–28]. Although relations between the extent of photobleaching and the phototoxic effects have, as yet, been poorly studied for KR, it is well established that photobleaching accompanies its photochemical reactions. The decrease of KR fluorescence intensity after exposure to light has been shown in cell cultures [1, 6, 11] and in tumor xenografts expressing this protein [8]. The most probable mechanisms of KR photobleaching are the direct reaction of molecular oxygen with the excited chromophore or the photoreduction of the chromophore owing to electron transfer from neighboring amino acid residues [4, 5]. Both processes can lead to the formation of a superoxide anion radical responsible for the phototoxicity of the KR.

In our study, the extent of photobleaching of KR in tumors was assessed for a range of light doses: 66–624 J/cm^2^, delivered using CW or pulsed lasers. It should be noted that the CW and pulsed lasers applied in this study have different wavelengths. The choice of 593 nm CW laser is explained by the discrete wavelengths of CW lasers available on the market, and this laser has the wavelength nearest to the maximum of KR absorption. In comparison to CW lasers, the tunable pulsed lasers allow selecting the specific wavelength within a wide range. Therefore, the wavelength of the tunable laser was chosen to be optimal, corresponding to the maximum absorption of KR. The absorption coefficient of KR at 593 nm is only 16% less than that at 584 nm that is not essential for the observed differences of tumor treatment in both regimes. Nevertheless, this difference partly explains that in the CW mode the light dose should be higher in order to obtain the same photobleaching effect as in the pulsed mode (Fig 2B).

One of the advantages of pulsed- over CW-irradiation is its reduced thermal effects. In classical PDT with CW light it is considered that, at fluence rates below 150 mW/cm^2^, heat generation is negligible and hyperthermia of the tissue can be avoided [12, 13]. The results of our study are consistent with this assumption. When a fluence rate of 150 mW/cm^2^ was applied, the temperature on the skin surface increased only by 2.1°C on average. At greater fluence rates tissue heating was more pronounced.

In an earlier study, the possibility of inducing cellular damage in HeLa tumors using KR with multiple CW irradiations at 270 J/cm^2^ was demonstrated [8]. In the present work, the more rapidly growing CT26 tumor was used, and the number of irradiation exposures was reduced from 7 to 3. However, the same light dose and mode failed to initiate any structural changes in the CT26-KR tumors. By contrast, in pulsed mode, the result was not only significant changes within the tumor cells, but also the inhibition of tumor growth. Possible explanations for this are that the higher light dose delivered to the tissue (337 J/cm^2^), and more effective photochemical reaction initiated by high-energy pulse, resulted in a higher photobleaching rate.

It is known that subcellular localization of the photosensitizer is of great importance in PDT as it determines to a large extent the mechanism a cell death and the overall cytotoxicity. As the cell nucleus is very sensitive to oxidative damage, we used KR expressed in fusion with histone H2B. Previously, various effects of nucleus-targeted KR have been demonstrated *in vitro*. For example, in the study by Serebrovskaya et al. a blockage of cell division was demonstrated owing to massive light-induced damage of the genomic DNA with the KR fused to histone H2B [6]. Waldeck et al. investigated the damaging effects of KR fused to the nuclear lamina and to histone H2A, on the basis of detection of DNA strand breaks [29]. By fusing KR to a tet-repressor or a transcription activator Lan et al. found that oxidative DNA damage occurs differently within hetero or euchromatin [30]. Correspondingly, as a consequence of KR-mediated specific DNA damage, a decrease in mitotic activity and an activation of apoptosis in the tumor cells were observed in our work.

However, localization of the photosensitizer is not the only factor affecting the mechanisms of cell death and the efficiency of PDT. The therapeutic outcomes of PDT are influenced to a large extent by the PDT dose regimen that includes the dose of light, the dose of photosensitizer and the drug-light interval. In the case of chemical sensitizers, the uptake and kinetics of the drug can differ for different tumors and this makes dosimetry rather complicated. In the case of KR, the stable expression of the protein in tumor cells allowed us to investigate, exclusively, the effect of light intensity and mode on the phototoxic manifestations of the protein.

It should be remembered that the cytotoxic efficiency of PDT using pulsed lasers strongly depends on the irradiation parameters, namely the repetition rate and the duration and energy of the pulse. There are the examples where pulsed laser-mediated PDT has appeared comparable [31–34] or inferior [35] to CW. The reasons for this may be the use of excessively high pulse energies at low repetition rates, resulting in saturation of the photosensitizer excited state, very high repetition rates leading to oxygen depletion, or the use of very short pulse durations, insufficient for pumping of the photosensitizer into the triplet state.

Nevertheless, several studies have also shown superior effects of PDT with pulsed lasers compared with CW if the above mentioned parameters are properly selected [36, 37, 38]. We suppose that better tumor responds to the pulsed mode in our research is associated with higher light dose delivered to the tissue (337 J/cm^2^ *vs* 270 J/cm^2^), more optimal wavelength of irradiation (593 nm *vs* 584 nm), and specific cellular disorders different from CW mode. In the context of comparison of CW and pulsed light, we should mention about the well-known effect of higher penetration of pulsed light into the tissue during PDT. Comprehensive theoretical analysis of the effectiveness of pulsed excitation in PDT by Sterenborg et al. [39] and the study of transient changes in light propagation performed on tissue-simulating media by Pogue et al. [34, 40] testify to the possibility of deeper penetration of the high-intensity pulsed irradiation in tissue than CW light by causing a transient decrease in the absorption of the photosensitizer during the time of the pulse. Apparently, this effect depends upon the optical properties of the tissues, the absorption due to the photosensitizer and the peak pulse irradiance, and can give significant increase in PDT depth *in vivo*, as demonstrated in Ref. [38]. However, we believe that in our study the effect of absorption saturation of KR is negligible because the contribution of KR in the overall light attenuation in tissue is very small (5% max). This is shown in the additional experiment using optoacoustic imaging (S1 File). Even if the effect of absorption saturation of KR is produced, the difference in light penetration depth for CW and pulsed irradiation should be insignificant. Saturation of the endogenous chromophores neither can contribute, as shown in Ref. [41], where the pulsed light did not have a greater depth of penetration than CW.

In Ref. [42, 43] a comparison of pulsed and CW laser modes for PDT with chemical sensitizers showed that irradiation using pulsed mode induced predominantly apoptotic cell death in monolayer cell cultures, while in the case of CW mode, the cancer cells underwent necrosis. Very recently, we demonstrated similar results on KR-expressing tumor spheroids [11]. This indicates the importance of appropriate selection of the treatment mode. Our results on KR-expressing tumors are in accordance with those findings: a more than 40-fold increase in the percentage of apoptotic cells was revealed when using pulsed mode.

## Conclusions

Therefore, in this work we developed an effective treatment regimen for PDT with the genetically encoded photosensitizer KR. The remarkable phototoxic effects of KR in mouse colorectal cancer CT26 were induced when using pulsed mode. The effects were manifested in a higher extent of photobleaching, pronounced histopathological abnormalities and slowing of tumor growth. However, since the average light doses and wavelengths were different for the pulsed and CW modes, direct comparison of their antitumor effectiveness is impossible in the scope of this work. The results of the study may be of interest not only for PDT but also for other fields of application of the phototoxic protein KR—optogenetics and light-induced protein inactivation.

## Supporting Information

S1 File Fluorescence and optoacoustic signals of KR. Measuring fluorescence and optoacoustic signals in CT26-KR tumor during pulsed laser irradiation (**Figure A**).
(DOCX)
