# Supplementary material for: Towards PDT with Genetically Encoded Photosensitizer KillerRed: A Comparison of Continuous and Pulsed Laser Regimens in an Animal Tumor Model
Source: PLoS One. 2015 Dec 11;10(12):e0144617. doi: 10.1371/journal.pone.0144617 (PMC4686120; doi:10.1371/journal.pone.0144617)
Supplement: S1 File — Measuring fluorescence and optoacoustic signals in CT26-KR tumor during pulsed laser irradiation (Figure A). (DOCX) [file pone.0144617.s001.docx]

**S1 File. Fluorescence and optoacoustic signals of KR**. Measuring fluorescence and optoacoustic signals in CT26-KR tumor during pulsed laser irradiation (**Figure A**).

In order to determine the contribution of KR in overall attenuation of light in tumor tissue, we have conducted the following experiment.

Balb/c mouse was inoculated s.c. with CT26-KR cells to generate tumor, as described in the paper. All the procedures on animals were approved by the Ethical Committee of the Nizhny Novgorod State Medical Academy (Russia). On the day 8^th^ of the growth the tumor was irradiated during 33 minutes by 15 ns laser pulses with 10 Hz repetition rate at 584 nm laser wavelength with 22 mJ/cm^2^ incident fluence. Optoacoustic microscope [P Subochev, A Orlova, M Shirmanova, A Postnikova, I Turchin // Simultaneous photoacoustic and optically mediated ultrasound microscopy: an in vivo study Biomedical optics express 6 (2), 631-638] was used to acquire 200 optoacoustic A-scans using 10 sec averaging period. The optoacoustic signal corresponding to 1 mm depth of the tumor was extracted from each A-scan and presented at Figure S1. During 33 minutes of pulsed laser exposure fluorescence signal dropped to 17% due to the photobleaching of KR. At the same time, optoacoustic signal, which is proportional to the optical absorption, dropped only to 95% at 1 mm depth of the tumor. As it is known from our previous study [S. Pletnev, et al. “Structural Basis for Phototoxicity of the Genetically Encoded Photosensitizer KillerRed”, J. Biol. Chem. 284(46), 32028–39, 2009], 80 % photobleached KR have approximately 4 time lower absorption coefficient than non-photobleached. Therefore, this result confirms that the tumor’s optical absorption provided by hemoglobin and other non-photobleachable endogeneous chromophores exceeds the optical absorption of photobleachable KR expressed in the tumor.


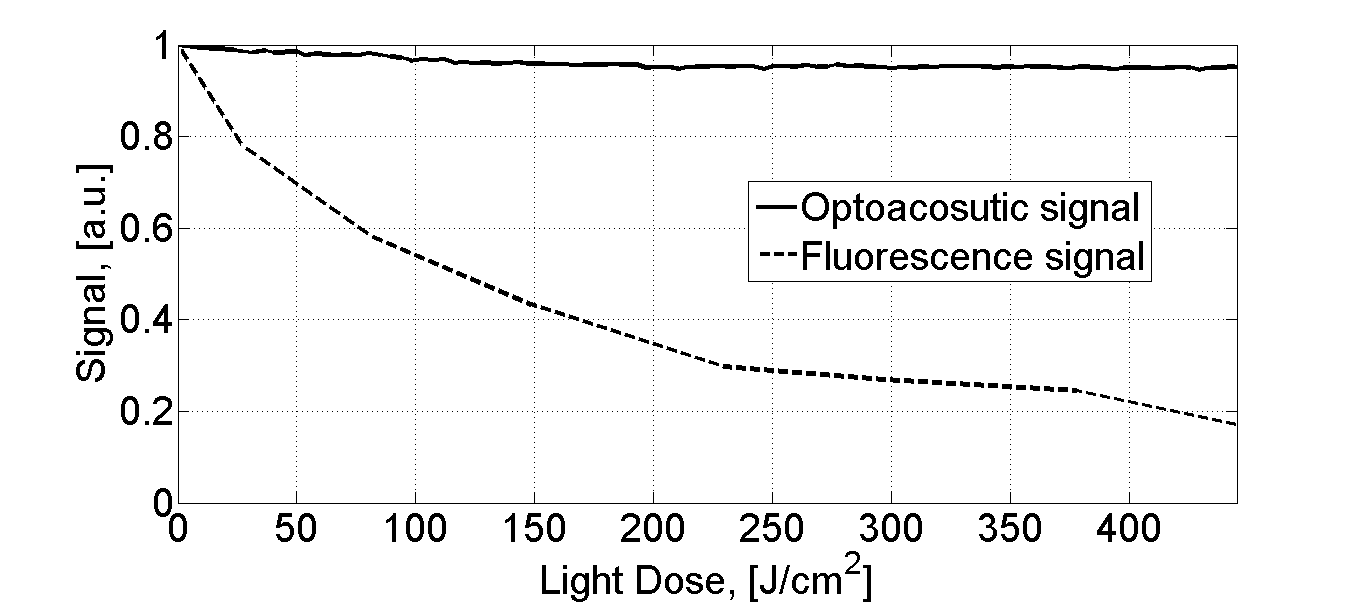


**Figure A.** Fluorescence and optoacoustic signals of KR in CT26-KR tumor during pulsed laser irradiation.
